# Will the Dental Colleges Reverse Their Decision?

**Published:** 1904-02

**Authors:** 


### Editorial.

### WILL THE DENTAL COLLEGES REVERSE THEIR DECISION?

TWENTY years in the past the organization known as the National Association of Dental Faculties was born, or, to be exact, August 4, 1884. From that time to the present year there has been a constant struggle to legislate for a higher standard of dental education in this country. Through a fifth of a century, step by step, the culmination of all these years of anxious labor was reached when the vote was finally announced in the Faculties Association that the resolution requiring that four years of seven months in each course had been adopted, and that all the colleges in membership with the organization were expected to faithfully comply with its demands. The unanimity of this vote was not the least pleasing feature of this advanced stand taken in dental education. It seemed to imply something more than a passing resolve that might possibly lead to a change from varied motives, but indicated that dentistry had made, practically, its final stand in the United States for thorough training, and from this decision there could be no retrograde movement tolerated.

The meeting preceding the enforcement of this resolution was that held at Asheville, N. C., in August last. Rumors were rife that this session would witness a change of opinion and possibly an effort might be made to rescind the former action. The vote, however, on the Harvard application, refusing permission for that school to remain at three years, seemed to settle all fears in that direction, in the minds of those present, as to the stability of the four years' course.

Three months subsequent to this meeting rumors were again heard that an effort was being made in certain quarters to call a meeting of the Association of Faculties at Buffalo during the session of the Institute of Pedagogics, to consider the necessity of returning to a three years' course. Even then it was not supposed that the active workers in this move would carry this out in violation of all precedent traditions, and in actual repudiation of established rules of the organization. Those who thus regarded the matter were doomed to disappointment, and were startled to receive a notice, in due form, from the chairman of the Executive Committee, that upon the demand of five members (colleges) belonging to the Association a meeting of the National Association of Faculties would be held in Buffalo at the time named. This meeting has since been held, and hence, whether for good or ill, its work has passed into history.

The fact that this was done cannot be lightly passed over. Dental education is the most important topic to claim the consideration of broad-minded men in dentistry, and to permit this attempt to lower its standard without journalistic comment would be to render valueless all periodical literature, for herein lies its especial duty.

This meeting must have been a disappointment to those who anticipated much from its decisions, for in this respect it was inconclusive, and this should have been anticipated before a single notice had been sent forth. If this were understood in advance of action, then those who engineered the meeting must have been fully cognizant that it would resolve itself into a conference—politically, a caucus—and thus crystallize sentiment to enable them to carry the backward movement with a hurrah at the annual meeting to be held the present year. With this motive, if held, the writer has nothing to do, for the importance of the main issue transcends all seeming sinister motives, for it strikes at the very root of dental education in this country, and, if successful, will place this where it stood twenty years ago. One retrograde step is simply the forerunner of another. This is the lesson of history, and must not be forgotten or be overlooked.

The immediate injury, however, while destructive to dental education in general, will be equally injurious to the association that represents it, and here the writer desires to dwell for the time being.

When persons join an organization, of whatever character, they become by both word and deed bound to obey the rules of said body, and under no circumstances, however tempting, should they even offer to violate these. For what, then, have these members become responsible in calling this meeting?

First, the Executive Committee has never been given power to call a special meeting of the Association of Faculties. No provision has ever been made in its rules for any such meeting, for its whole spirit since its origin has been adverse to the possibility of a snap judgment upon vital questions. No such meeting in its entire history was ever contemplated or desired. The article in the constitution giving that committee power to call the regular meeting at "such time and place" as might be deemed necessary was a wise provision to give liberty to change in case the Executive Committee of the National Dental Association selected a different time and place of meeting for that body, a change frequently found desirable. The intention was that the Faculties should always meet at the same time and place with this body. This was done largely as a matter of convenience for those members who belonged to both organizations, to avoid unnecessary travel and expense. The only time in the history of the Faculties that it met independently was at Chicago in 1885, when the then national body, the American Dental Association, met at Minneapolis. This proved the least valuable meeting of the entire series, and this was altogether due to the fact that the work was hurriedly passed upon by the members, so that they could proceed to the aforesaid national gathering. Some of those present at these Chicago sessions have been in opposition to any meeting separate from the national body since that day.

The present meeting was called at the request of five colleges. As before stated, there is no authority for this. It is presumed some had been reading the report of the Committee on Codification of the Rules, presented at the Asheville meeting, and in which the said committee interpolated a provision for the calling of special meetings, but, as this report was laid over for action at the regular meeting in 1904, it has no force until its adoption, and therefore the Executive Committee exceeded its powers in consenting to the request of the five members (colleges). In the call for special meetings in all organized bodies, when these have rules providing for them, it is necessary to state in the call the subjects to be acted upon, and this was not neglected upon this occasion, and it was, perhaps, the only regular part of the proceedings. The members were fully notified that the "Four-Year Rule," together with the "Count" question, would be considered. It must have been well known that the first had no legal status in such a meeting, for all subjects affecting the interests of dental colleges connected with the Association must lie over for one year before action, and due notice must be given to this effect in the official report. This applies with equal force to the second proposition. This being understood, it is not surprising that the special meeting resolved itself into a discussional gathering and resulted in a fiasco, as far as dealing finally with the problems named. From what has been gathered from reports, not much comfort was derived for those who advocated a course of three years with nine months in each session, for the general spirit was antagonistic to even this compromise. There seemed to be an entire want of harmony of purpose with those who desired retrograde action.

The writer feels that a wrong has been done the Association of Faculties, first, by calling this meeting without authority, and, second, in the attempt to lower the standard of dental education it had so carefully secured through years of constant struggle; and, further, in thus attempting a retrograde they have tried, whether intentional or not, to make dental education in this country a subject for reproach throughout the professional world.

Will this effort to effect a return to three years be successful? The answer to this question can best be given after the close of the next regular meeting of the Faculties. That the effort will be made by the colleges interested in this retrograde movement is self-evident, but it is not possible that it can be successfully carried through. The writer has not lost faith in that professional spirit that looks to higher results than the sordid accumulation of money. It is believed that there are a sufficient number who will stand solidly for the advanced standard already established. If, however, this trustful feeling should not be realized, and the next meeting should show that the National Association of Dental Faculties had reversed its position on the question of dental education, then may the dental profession in the United States mark the beginning of decadence of the professional character so earnestly striven for during the past half-century, and, in addition, it will present a spectacle for a jeering professional world. May our colleagues seriously consider the situation, and come prepared at the annual meeting to uphold, at all hazards, the honor of American dentistry.

It was suggested at the Buffalo, so-called, special meeting that the Faculties be called together in June instead of August, possibly at Louisville, Ky. In view of what has been already stated, this is simply a usurpation of power on the part of the Executive Committee, for the meeting, as organized, had not the slightest authority to change the original intention of meeting at St. Louis. It is to be hoped that the Executive Committee will not fall twice into error in one year. It must be remembered that probably all the members will desire to visit St. Louis during the Exposition and the World's Dental Congress. This membership extends from New England to California, and it is unreasonable to ask these widely separated members to travel long distances in summer, in some cases across the continent, to attend two meetings. The experiment of holding a separate meeting has already been considered, and the injurious effect demonstrated then will be intensified during this year if this be attempted. The men who will actively manage the World's Dental Congress are those mainly interested in dental education, and their interests should be considered. Those who will attend the Congress at St. Louis will naturally arrange to spend several weeks there. This was the experience of those in attendance at the Columbian Exposition at Chicago, and no difficulty was found then in holding the meetings in advance of the great world's dental convocation. It is very true that the success of this must be assured regardless of what may be termed local interests, but if it is desired to injure the Congress, there seems no better way than to decentralize the various meetings of the year. A much better plan would be to postpone all the national meetings until 1905, for nothing would suffer by this action, while it would concentrate effort on the one meeting that should and doubtless will represent the dentistry of the world as it exists in 1904.

